# Calprotectin as a New Sensitive Marker of Neutrophilic Inflammation in Patients with Bronchiolitis Obliterans

**DOI:** 10.1155/2020/4641585

**Published:** 2020-05-01

**Authors:** S. P. Jerkic, F. Michel, H. Donath, E. Herrmann, R. Schubert, M. Rosewich, S. Zielen

**Affiliations:** ^1^Department for Children and Adolescents, Division of Allergology, Pulmonology and Cystic Fibrosis, Goethe University, Frankfurt, Germany; ^2^Institute of Biostatistics and Mathematical Modeling, Goethe University, Frankfurt, Germany

## Abstract

**Introduction:**

Bronchiolitis obliterans (BO) is a chronic disease in which persistent inflammation leads to obstruction and obliteration of the small airways. The aim of this study was to evaluate the value of calprotectin as an inflammatory marker in induced sputum.

**Methods:**

Twenty-eight patients suffering from BO and 18 healthy controls were examined. Lung function was measured by spirometry, body plethysmography, and lung clearance index (LCI). The induced sputum was obtained, cell counts were performed, and cytokines were measured using cytometric bead array (CBA). Calprotectin was quantified in the sputum and serum samples using commercially available sandwich ELISA.

**Results:**

Spirometry parameters including forced vital capacity (FVC), forced expiratory volume in 1 second (FEV1), and maximum expiratory flow rate at 25% vital capacity (MEF25) were significantly lower in BO patients than in healthy controls, whereas the reserve volume (RV), RV to total lung capacity ratio (RV/TLC), and LCI were significantly increased. In sputum, calprotectin levels, neutrophils, and IL-8 were significantly elevated. Calprotectin levels correlated strongly with IL-8 and other biomarkers, neutrophils FEV1 and MEF25. In serum, calprotectin was significantly diminished in BO patients compared to controls.

**Conclusion:**

Lung function is severely impaired in BO patients. Calprotectin is significantly elevated in the sputum of BO patients and reflects ongoing neutrophilic inflammation.

## 1. Introduction

Bronchiolitis obliterans (BO) is a rare, chronic form of obstructive lung disease. Most early descriptions of BO were limited to case reports with autopsy findings. Typically, BO begins with an injury to the bronchiolar epithelium followed by an inflammatory reaction that progresses to airway fibrosis and potential luminal obliteration [1, 2]. The initial severe insult is thought to affect the lower airways and can be caused by either a pathogen, postinfectious BO (PIBO), lung transplantation (LT) or bone marrow transplantation (BMT), or BO syndrome (BOS) [3–5]. This failure of resolution of initial and ongoing inflammation is likely to be an important part of the disease process of BO. There is an influx of inflammatory and immune cells. In combination with the proliferation of granulation tissue in the small airways, this leads to obstruction of the bronchioli with air trapping and hyperinflation [1, 2, 6].

BO is defined clinically as a small airway disease with persistent air flow obstruction and air trapping as determined by lung function measurements. In general, this airflow obstruction is nonresponsive to bronchodilators. Most studies use spirometry to measure lung function in BO, but current guidelines highlight the need for new sensitive lung function indices to detect early small airway impairment. In the last decade, the lung clearance index (LCI) has repeatedly been shown to be superior to the forced expiratory volume in 1 second (FEV1) to monitor small airway disease in different lung diseases. The LCI is a feasible and sensitive tool to detect small airway impairment in chronic lung diseases in children and adults [7–10]. To the best of our knowledge, only a few studies have used this technique in patients with BO [11, 12].

Previous investigations have shown that BO is predominantly a neutrophilic disease of the small bronchioles featuring elevated levels of proinflammatory cytokines [5]. Analysis of the sputum of BO patients reveals consistent neutrophil inflammation, while the levels of inflammatory cytokines such as IL-1*β*, IL-6, and IL-8 have greater variability [13]. As progression patterns have not yet been defined and due to the predominantly persistent neutrophilic inflammation in a changing inflammatory pattern, calprotectin, which is found extensively in the cytoplasm, might serve as a valuable surrogate biomarker for BO.

Calprotectin is a 36 kDa zinc-binding protein that is universally found in the body and in the cytoplasm of neutrophils and macrophages. Calprotectin is released from activated neutrophils, leading to high concentrations in various fluids, such as plasma, serum, spinal fluid, synovial fluid, urine, saliva, or stool [14]. Therefore, calprotectin is increased predominantly in inflammatory processes in relevant organs. As calprotectin is released locally, it may be a sensitive indicator of local inflammation [15]. Calprotectin is known as an excellent surrogate biomarker for the inflammatory status of the gut in inflammatory bowel disease. Calprotectin is measured in stool samples as there is an influx of inflammatory cells in the inflamed gut tissue in IBD, and it is known to be correlated with disease activity [14, 16].

This study investigates the value of calprotectin and cytokines in the sputum of BO patients. Additionally, this study explores the value of calprotectin as an inflammatory marker and its correlation with cytokine levels and LCI.

## 2. Methods

### 2.1. Patients and Controls

We prospectively studied twenty-eight patients with BO, including 21 patients with PIBO and 7 patients with BOS following human stem cell transplantation (HSCT). The median age of the patient cohort was 14.2 (6.2-27.3) years. The diagnosis BO was made by the following criteria: medical history of infectious insult or BMT, persistent crackles on clinical examination, lung function with an FEV1 < 75% and reversibility of less than 12%. In addition, most of the patients presented with typical radiological features at the time of diagnosis. Other causes of chronic respiratory symptoms, such as cystic fibrosis (CF), immunodeficiencies, aspiration syndrome, or congenital heart disease, were excluded in all patients. In addition, 18 healthy control subjects with a median age of 16.2 (7.6-25) years were examined. Written consent from patients or caregivers was required for every subject. The study followed the ethical principles of the Declaration of Helsinki, the regulatory requirements, and the code of Good Clinical Practice. The study was approved by the responsible ethic committee at the Frankfurt University and registered at clinicaltrials.gov (NCT02627833).

### 2.2. Lung Function Tests

The lung function tests and reversibility testing were evaluated using a body plethysmograph (VIASYS Healthcare GmbH, Hoechberg, Germany). The following parameters were recorded: forced vital capacity (FVC), FEV1, FEV1/VC, 75% of the maximum expiratory flow (FEF 75%), reserve volume (RV), and the ratio of RV to total lung capacity (RV/TLC). The LCI was measured by EasyOne Pro Lab (ndd Medical Technologies, Andover, Massachusetts, USA).

Lung function tests (spirometry and body plethysmography) and the LCI were evaluated in all patients at routine clinical visits. If the patients were receiving beta-agonists, they were advised to discontinue administration 8 hours prior to testing, and long-acting beta-agonists were to be withdrawn 24 hours before the study. FVC, FEV1, FEF 75%, and flow-volume curves were evaluated according to the American Thoracic Society (ATS) guidelines. Lung volumes (TLC, forced residual capacity (FRC), and RV) were measured by plethysmography following international recommendations [17].

### 2.3. Airway Reversibility

Bronchodilator reversibility was tested after inhalation of 400 *μ*g of salbutamol by a metered dose inhaler (MDI) with a spacer. Reversibility was defined as yes/no using a ≥12% and 200 ml post bronchodilator change.

### 2.4. Sputum Collection and Processing and Cell Analysis

The patients and control subjects first inhaled salbutamol followed by nebulization with hypertonic saline solution at increasing concentrations of 3%, 4%, and 5% every 7 minutes, as recently described [18, 19]. The sputum was processed within 1 hour of collection. The sputum was quantified, and sputum plugs were selected from the samples and placed in preweighted Eppendorf tubes. They were treated with 4x weight/volume 0.1% dithiothreitol (DTT), and the samples were processed for 15 minutes on ice before the subsequent addition of 2x the weight/volume of phosphate-buffered saline (PBS). Samples were filtered through a 48 *μ*m mesh and centrifuged for 10 minutes at 790 × g. Supernatants were stored at -80°C until further analysis. The pellet was resuspended in 300 *μ*l of 2% PBS/BS. Subsequently, the mix was set to 30,000 cells/ml for cytospin analysis, which was stained with May-Grunwald-Giemsa for differential cell counting [18, 19]. In total, 400 cells per slide were counted, and the percentages of neutrophils, lymphocytes, eosinophils, and macrophages were expressed as proportions of the total cell count.

### 2.5. Cytometric Bead Array (CBA)

The concentrations of three cytokines were determined in sputum samples using the BD™ CBA Flex Set System (BD Bioscience-PharMingen, San Diego, CA, USA) to measure IL-6, IL-8, and IL-1*β* levels. Each BD™ CBA Flex Set contained a one-bead population with distinct fluorescence intensity and both the appropriate phycoerythrin (PE) detection reagent and the standard. The tests were performed according to the manufacturer's instructions. To analyse the cytokines, we added the same concentration of DTT (0.025%) as in the sputum supernatant to the standard curve and enzyme immunoassay buffer as described [20]. The lower detection limits of the cytokines were 1.2 pg/ml, 1.6 pg/ml, and 1.0 pg/ml for IL-8, IL-6, and IL-1*β*, respectively.

### 2.6. Statistics and Data Analysis

Data were analysed using GraphPad Prism (GraphPad Software Inc., La Jolla, CA, USA) and Microsoft Excel. Group differences between BO patients and control subjects were analysed using the Kruskal-Wallis test or Mann–Whitney *U* test depending on the normality and homogeneity of variance assumptions. A probability of *p* < 0.05 was considered significant.

## 3. Results

As shown in Table 1, we enrolled 28 patients (median age, 14.2; range, 6-27; male/female ratio, 11/7). We compared the patients' data with those of 18 healthy control subjects (median age, 16.2; range, 7-25; male/female ratio 16/12) (Table 1).

### 3.1. Lung Function Test Results

Patients with BO revealed significantly compromised lung function testing. The FVC, FEV1, and the maximum expiratory flow rate at 25% vital capacity (MEF25) were significantly decreased, while RV/TLC and LCI were significantly increased in BO patients (BO: 12.1, 7.3-20.5; controls: 7.1, 6.0-8.4, *p* < 0.001) compared to controls (Table 1).

### 3.2. Airway Reversibility

**Figure 1:**
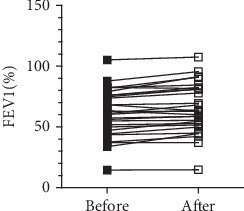
Jerkic et al.

No airway reversibility was detected in the majority of BO patients (Figure 1). Airway reversibility testing after administration of 400 *μ*g of salbutamol was positive in only one patient with an FEV1 increase of >12% and >200 ml and in three patients with FEV1 increases of >12% but <200 ml.

### 3.3. Analysis of Inflammatory Cells and Cytokine Levels in Induced Sputum

**Figure 2:**
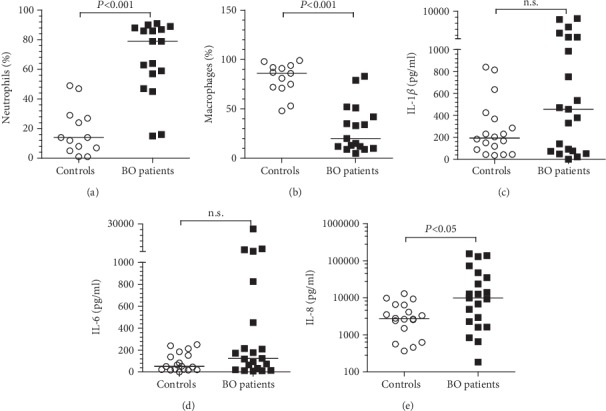
Jerkic et al.

As shown in Figure 2, a significantly higher percentage of neutrophils (BO patients: 79%, 15-91%; control subjects: 14%, 1-49%, *p* < 0.001) was observed in all the BO patients, whereas the control subjects showed predominantly alveolar macrophages (BO patients: 20%, 5-83%; control subjects: 86%, 48-99%, *p* < 0.001) in their sputum samples.

All tested cytokines were detected in the sputum supernatants of BO patients and 19 control subjects. As shown in Figure 2, the levels of IL-1*β*, IL-6, and IL-8 were higher in the BO patients than in the controls, but only IL-8 levels (BO: 9855 pg/ml, 182-155228 pg/ml; controls: 2734 pg/ml, 366-13018 pg/ml, *p* < 0.05) were significantly different.

### 3.4. Serum and Sputum Calprotectin Levels

**Figure 3:**
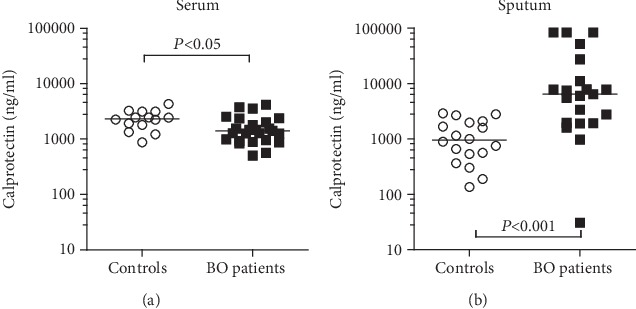
Jerkic et al.

Serum calprotectin concentrations (BO patients: 1416 ng/ml, 504-4161 ng/ml; control subjects: 2342 ng/ml, 875-4331 ng/ml, *p* < 0.05) were significantly lower in BO patients than in control subjects (Figure 3(a)).

In contrast, sputum calprotectin levels were significantly elevated in BO patients compared to control subjects (BO patients: 6429 ng/ml, 30.6-84000 ng/ml; control subjects: 950 ng/ml, 135-2887 ng/ml, *p* < 0.001) as shown in Figure 3(b).

There was a significant correlation of calprotectin with IL-8 levels (rho = 0.871; *p* < 0.0001) and of calprotectin with neutrophil counts, FEV1, MEF25, and the LCI (Table 2). There was no significant correlation of calprotectin with IL-1*β* and IL-6.

## 4. Discussion

The term “bronchiolitis obliterans” describes common pathological alterations in the small airways following a variety of inciting diseases with different aetiologies and characteristics [1, 2]. The process that leads to the observed pathology in the small airways is influenced not only by the initial insult but also by the localized inflammatory response and preexisting factors, such as nutritional status and genetic variants [2–4]. Previous studies have demonstrated an ongoing neutrophilic inflammation and increased proinflammatory markers, especially IL-8 in the airways of patients with BO [5, 13, 21]. Nevertheless, there is an ongoing search for biomarkers that can be used to assess the risk and disease activity in a single patient. Calprotectin is a protein that is found extensively in the cytoplasm of neutrophils. It is released passively after cell death within the limits of an inflammatory process, and its value in inflammatory bowel disease is well established [14–16]. As expected, calprotectin was significantly increased in the sputum samples of the BO cohort compared to the control subjects. In addition, there was a significant correlation of calprotectin with neutrophil counts, IL-8 levels, and the levels of the proinflammatory cytokines IL-1*β* and IL-6. These results suggest that calprotectin could be a reliable indicator of inflammatory processes within the small airways. The most significant correlation was found between IL-8 and calprotectin. IL-8 is produced by epithelial cells and has a chemotactic effect on neutrophils. The increase in both markers, IL-8 and neutrophils, strongly suggests persistent inflammation in the small airways in our BO cohort. Since our protocol excluded BO patients with acute respiratory infections and because some of them were already diagnosed with BO more than 10 years ago, it is very likely that there is an underlying chronic inflammatory process rather than inflammation triggered by an acute infection.

Michel et al. [22] investigated induced sputum in healthy control subjects before and 24 hours after lipopolysaccharide (LPS) inhalation and showed a correlation between increased neutrophil counts and calprotectin levels in the sputum. Repeated measurements showed reproducibly increased calprotectin levels; however, increased neutrophil counts were not correlated with the results of the first attempt [22]. This finding could indicate that there is an advantage in terms of the reproducibility of calprotectin levels compared to neutrophil counts in sputum. Studies by Gray et al. [23] suggested that calprotectin could indicate changes due to inflammatory processes. Calprotectin levels measured in the sputum of CF patients decreased significantly after antibiotic treatment for an acute exacerbation [23, 24]. However, further investigations are necessary to reveal whether calprotectin levels measured in the sputum of BO patients decrease equally after antibiotic and anti-inflammatory treatments.

Further investigations in patients with CF have determined that increased serum calprotectin levels reflect inflammatory activity [25]. Presumably, increased serum calprotectin levels could occur in circulation either by leaky epithelial barriers at the site of inflammation or by increased recruitment of neutrophils from the bone marrow [24]. However, in our study, there was no increase in calprotectin levels in the serum samples of the BO cohort but a clear reduction in serum calprotectin levels. These results in BO patients may be because BO is associated with local inflammation that cannot be captured by systemic calprotectin levels; therefore, serum calprotectin is not a useful marker for airway inflammatory activity in BO. As an inflammatory marker, calprotectin has been well established in IBD [15, 16]. Testing calprotectin levels is inexpensive, reproducible, and available. Therefore, calprotectin in the sputum could be used as an effective biomarker to identify patients with suspected diagnoses of BO. In addition, it would be helpful to investigate further whether there is an equally qualified method to measure sputum calprotectin levels that is not as time intensive as the present method.

Patients with BO typically develop small airway disease, which presents with significant flow limitations particularly affecting the small airways and typical signs of air trapping in distal areas of the lung [2–5]. Previous studies have revealed that there is a correlation between obstruction of the small airways and the neutrophil counts in the sputum and bronchoalveolar lavage (BAL) fluid. There is a clear relationship between persistent neutrophilic inflammation and small airway disease in patients with BO, CF, and COPD [5, 21, 26–28]. There is evidence in CF that the LCI detects changes within the small airways much more sensitively than other tests [9]. The LCI in our BO cohort was significantly increased, and there was considerable inhomogeneity of ventilation, which is due to obstruction of the small airways. A significantly increased RV and RV/TLC and a decreased FEF 75 confirmed this finding. In addition, we found a significant correlation between the LCI and FEF 75, which suggests that both parameters characterize the extent of obstruction of the small airways. Previous studies have shown a continuous increase in the inhomogeneity of ventilation in patients with BOS measured by the LCI before any significant decline in spirometry was found [11, 12]. Lahzami et al. [11] suggested that regular LCI measurements in patients after HSCT and LT could show the early stages of BOS; therefore, early treatment options could be established to prevent further fibrotic changes. However, further longitudinal measurements of the LCI are required to show whether the LCI is a sensitive and effective measure of the early stages of BO. In this study, there was a significant correlation between lung function test results and sputum calprotectin levels. A decreased FEV1 and FEF 75 and an increased LCI were clearly correlated with increased calprotectin levels in sputum samples from the BO cohort. BO patients with higher levels of calprotectin, indicating intensified inflammatory activity, showed worse obstruction in terms of lung function test results. However, there was no correlation between hyperinflation (RV and RV/TLC) and calprotectin levels.

## 5. Conclusion

BO is associated with chronic inflammation of the small airways that leads to obstructive airway disease. Due to persistent predominantly neutrophilic inflammation, calprotectin, which is released by neutrophils, is significantly increased in the sputum of patients with BO. Further investigations are required to confirm whether calprotectin is a sensible surrogate marker of neutrophilic inflammation to support the diagnosis of BO and to monitor the effects of anti-inflammatory therapy in BO.

## Figures and Tables

**Table 1:** Patient characteristics.

	Controls	Patients with BO	*p* value
*n*	18	28	—
Age (years)	16.2 (7-25)	14.2 (6-27)	n.s.
Sex (m/f)	11/7	16/12	n.s.
FVC (%)	93.0 (71.6-105.8)	80.7 (25.5-107.4)	<0.001
FEV1 (%)	97.9 (83.3-114.9)	61.9 (14.6-105.2)	<0.001
MEF25 (%)	103.1 (44.9-137.1)	26.7 (6.7-107.9)	<0.001
RV/TLC (%)	134.8 (82.0-153.2)	177.3 (117.2-300.4)	<0.001
LCI	7.1 (6.0-8.4)	12.1 (7.3-20.5)	<0.001

**Table 2:** Correlations with calprotectin.

	Correlation coefficient (rho)
Neutrophils	0.550^∗∗^
IL-8	0.879^∗∗∗^
FVC	-0.173
FEV1	-0.445^∗∗^
MEF25	-0.445^∗∗^
RV/TLC	0.245
LCI	0.386^∗^

## Data Availability

All data of this paper are available on request. Please contact for this purpose Mrs Pera-Silvija Jerkic (Pera-Silvija.jerkic@kgu.de).

## Additional Points

*Key messages.* What is the key question. Can calprotectin, which is a well-established and locally released marker, be used as sensitive indicator for persistent neutrophilic airway inflammation in patients with bronchiolitis obliterans? What is the bottom line. Calprotectin levels in sputum were significantly increased in patients with bronchiolitis obliterans (BO) and correlated strongly with IL-8 and an increased lung clearance index. Why read on. Elevated calprotectin levels reflect persistent neutrophilic airway inflammation, and calprotectin is a new and reliable biomarker in BO.
